# What Is the Most Reliable Concordance Rate of Preoperative Synovial Fluid Aspiration and Intraoperative Biopsy to Detect Periprosthetic Joint Infection in Knee, Hip and Shoulder Arthroplasty?

**DOI:** 10.3390/antibiotics12101482

**Published:** 2023-09-25

**Authors:** Mustafa Akkaya, Luigi Zanna, Rudy Sangaletti, Ali Bokhari, Thorsten Gehrke, Mustafa Citak

**Affiliations:** Department of Orthopaedic Surgery, Helios ENDO-Klinik Hamburg, 22767 Hamburg, Germany; makkaya@aybu.edu.tr (M.A.); luigi.zanna@unifi.it (L.Z.); rudy.sangaletti@poliambulanza.it (R.S.); syed.bokhari@health.nsw.gov.au (A.B.);

**Keywords:** arthroplasty, infection, synovial fluid, intraoperative biopsy

## Abstract

The accuracy of preoperative synovial fluid microbe detection in periprosthetic joint infection (PJI) is widely reported. However, the reliability of this diagnostic modality amongst the different joints is not yet described. We aimed to compare the concordance rate between preoperative synovial fluid and intraoperative tissue cultures in shoulder, knee and hip PJIs. A total of 150 patients who met the 2018 International Consensus Meeting criteria for shoulder, hip and knee PJI were retrospectively reviewed. This cohort was divided into three groups based on the involved joint (should, hip or knee), with 50 patients in each group. Cultures were collected and held for culture for 14 days. The overall concordance rate was 56.7%. Concordance rates between preoperative and intraoperative cultures were 60%, 56% and 54% for the knee, shoulder and hip joints, respectively. The analysis of high- or low-virulence and difficult- or not-difficult-to-treat germs did not reveal any significant differences between preoperative and intraoperative cultures in any of the groups. However, even considering the higher concordance in knee PJI, the overall discordance between preoperative and intraoperative cultures should prompt surgeons not to rely solely on preoperative synovial fluid culture data in determining appropriate treatment and antibiotics.

## 1. Introduction

The early, timely and accurate diagnosis of periprosthetic joint infection (PJI) is of utmost importance for treatment. Delays in diagnosis and treatment may result in incorrect treatment, treatment failure, prolonged hospital stay, seeding of the infection to other sites, time lost from work and psychosocial factors affecting both the surgeon and the patient [1].

Despite the increasing rates of PJI, making a diagnosis is still challenging due to the lack of an early sensitive and accurate test that can lead to a diagnosis. Currently, the 2018 International Consensus Meeting (ICM) modified Musculoskeletal Infection Society (MSIS) definition is widely used for PJI diagnosis. This definition is based on a combination of clinical findings, peripheral blood and synovial fluid samples, histologic evaluation and intraoperative findings.

Although serological tests and radiographic evaluation are important tools in an orthopedic surgeon’s armamentarium [2], the identification of pathogenic microorganisms is the key to accurate diagnosis and permits the administration of targeted antimicrobial treatment to maximise the chances of a successful outcome [3]. However, approximately 20–50% of patients have negative cultures despite clear clinical and laboratory evidence of PJI, giving rise to a diagnostic conundrum known as “culture-negative PJI” [4].

Preoperative synovial fluid cultures have several limitations due to low sensitivity, low bacterial loads found in low-grade PJI, presence of pathogens adherent on biofilm and technical issues such as delayed specimen handling or inadequate culture processing [5]. Misdiagnosis of the pathogen based on the preoperative culture may be responsible for improper antibiotic therapy and surgical planning and possible treatment failure [6].

Despite this, the preoperative synovial fluid culture is still an important tool in the hands of both surgeons and infectious disease specialists because it can guide early treatment, as final results of intraoperative tissue cultures cannot be obtained in the early postoperative period [7]. This information is of vital importance for the treatment regimen, and a successful PJI treatment can usually be achieved when preoperative and intraoperative results are concordant, in contrast to the setting of discordance between preoperative and intraoperative results where a correct targeted treatment plan can become challenging and delayed [8,9].

Preoperative microbiological diagnosis is the main condition of treatment in patients with PJI who are planned to undergo one-stage septic exchange [10], whereas two-stage revision is recommended for patients with culture-negative PJI [11].

Therefore, concordance between microbiological culture methods is of utmost importance for clinics using the one-stage septic exchange treatment method. Some studies demonstrated a range of concordance between 52 and 78% between preoperative aspiration and intraoperative culture [5,12,13,14,15].

This study aimed to answer the following clinical questions: (1) What is the concordance rate between preoperative synovial fluid aspiration and intraoperative biopsy used in making a PJI diagnosis? (2) Is the said concordance rate different between the knee, hip and shoulder PJIs? Furthermore, we aimed to assess if there were different bacterial patterns between the joints, as this may alter the standard empirical therapies for each joint.

We hypothesized that the rate of concordance between preoperative and intraoperative cultures among the different joints (knee, hip and shoulder) is different and that preoperative cultures should not be relied upon to guide long-term treatment.

## 2. Results

**Figure 1:**
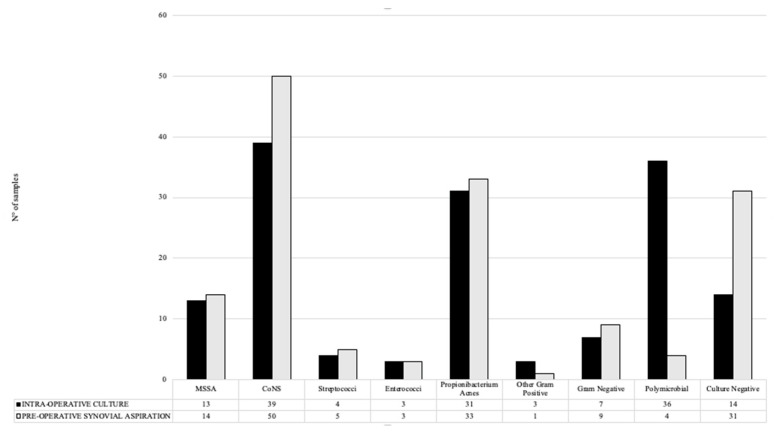
Pathogens detected in pre- and intraoperative cultures. MSSA: methicillin-sensitive *Staphylococcus Aureus*, CoNS: coagulase-negative *staphylococci*.

This study included a total of 150 patients divided into three groups (knee, shoulder and hip), each consisting of 50 patients. A total of 80 patients were male and 70 patients were female, with a mean age of 69.21 ± 11.71 years. The bacteria detected in preoperative synovial aspiration and intraoperative cultures are shown in Figure 1.

Preoperative aspiration and intraoperative biopsy cultures were found to be concordant in 85 of 150 (56.7%) patients and discordant in 65 (43.3%) patients. *Staphylococci* (52 patients) and coagulase-negative *staphylococci* (*CoNS*) (39 patients) were the most frequently intraoperatively isolated types of bacteria.

The analysis of patient demographics revealed no statistically significant differences in sex, in BMI and among PJI cases between concordant and discordant groups (Table 1). *Staphylococci* bacteria cultures showed a higher rate of concordance when compared to the discordant group (*p* = 0.0001), and similarly, *CoNS* demonstrated a similar difference with a significantly higher concordance rate (*p* = 0.0004). However, no significant differences between the two groups were reported for methicillin-sensitive *Staphylococcus Aureus* (*MSSA*). With regard to Gram-negative pathogens, a high rate of concordance (100%) was observed, reaching statistical significance *p* = 0.019 (Table 1). Within the discordant group, there was a significantly higher number of patients with polymicrobial PJI (34/36, 94.4%; *p* < 0.0001); see Table 1.

Evaluation of the hip, knee and shoulder groups separately did not yield a statistically significant difference in terms of age, BMI, sex and side of the affected extremity (Table 2). The concordance rate of intraoperative and preoperative aspiration cultures was 54% in the hip group, 60% in the knee group and 56% in the shoulder group. There was no statistically significant difference between the groups (Table 2). Comparison of the groups in terms of bacterial virulence (high and low virulence) in intraoperative cultures did not yield a significant difference between the knee and hip groups. In contrast, the rate of low-virulence bacteria in both preoperative and intraoperatively cultures was significantly higher in the shoulder group compared to both the knee and hip groups (*p* < 0.05) (Table 3). The groups were also compared in terms of difficult-to-treat bacteria in intraoperative cultures; there was no difference between the knee and hip groups, but a significantly lower rate of difficult-to-treat bacteria was demonstrated in the shoulder group (*p* < 0.05) (Table 3).

In comparing the preoperative and intraoperative cultures in each group, we reported a significantly higher number of culture-negative (CN) preoperative cultures than intraoperative in the shoulder group (*p* = 0.003), and we registered a significantly higher rate of polymicrobial intraoperative cultures than preoperative in the hip group (*p* = 0.48); see Table 4.

**Figure 2:**
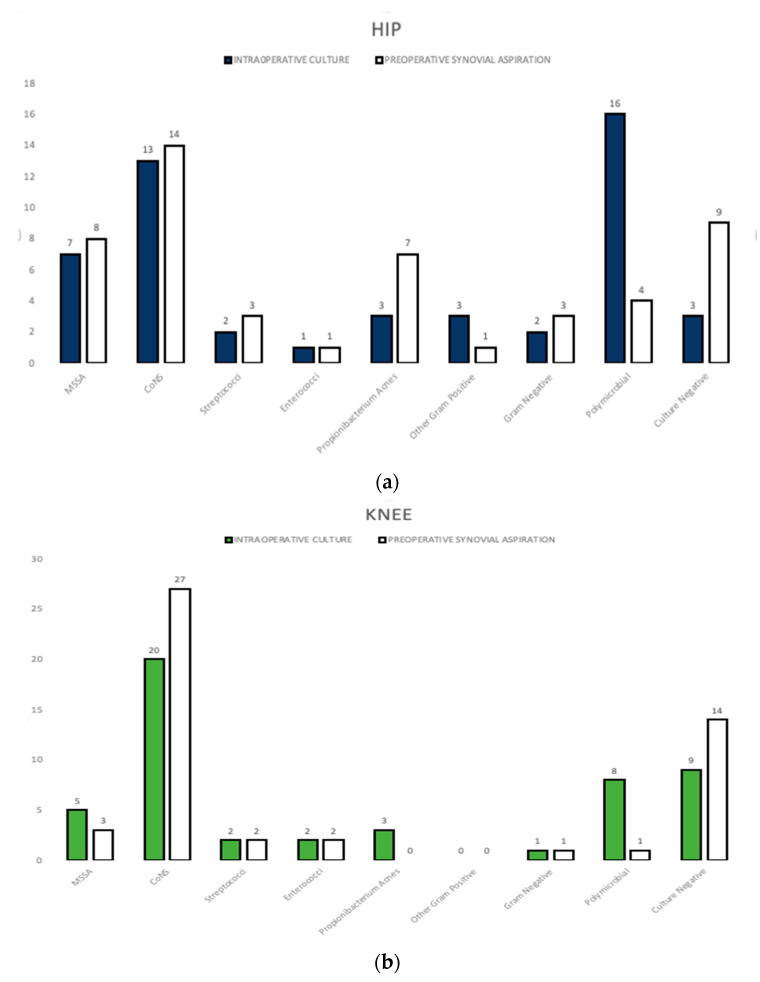

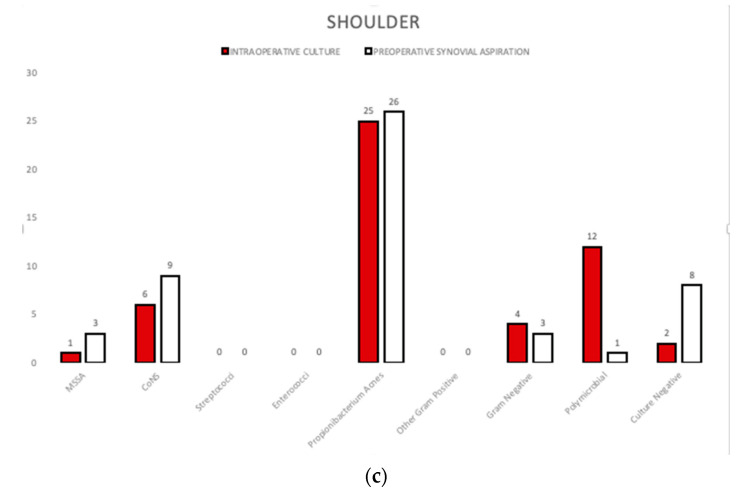
Pathogens detected in pre- and intraoperative cultures in the (**a**) hip, (**b**) knee and (**c**) shoulder groups. MSSA: methicillin-sensitive *Staphylococcus Aureus*, CoNS: coagulase-negative *staphylococci*.

The most commonly isolated bacterium in intraoperative and preoperative cultures was *CoNS* in the hip and knee groups and *Propionibacterium Acnes* in the shoulder group. Microbiological results according to groups are provided in detail in Figure 2.

## 3. Discussion

This study investigated the microbiological concordance between preoperative synovial fluid aspiration and intraoperative biopsy cultures in patients with PJI of the knee, hip and shoulder. To the best of our knowledge, this is the first study that analysed the difference in concordance rates between preoperative and intraoperative cultures in different joints affected by PJIs. In all groups, preoperative and intraoperative culture results were found to be concordant in more than half of the patients; there was a slightly higher concordance rate in the knee group (60%) than in both the hip and shoulder groups, though no significant statistical difference was demonstrated between the joints.

The higher rate of knee culture concordance compared to hip may be explained by the complexity of a hip aspiration. In hip aspiration, there is a significantly larger and thicker soft tissue envelope that must be traversed by a needle to reach the fluid; this challenge may result in a lower yield of fluid to subsequently culture and therefore less possible sensitivity [16]. Furthermore, the lower concordance rate of the shoulder compared to the knee, even though it was minimal, may be explained by the higher rate of low-virulence bacteria in the shoulder [17], and this may lead to a misdiagnosis of the pathogen and false-negative cultures. This is supported in our study, as the rates of high-virulence and difficult-to-treat bacteria were found to be significantly lower in the shoulder group compared to the hip and knee groups.

According to the literature, while preoperative aspiration culture is essential for a PJI diagnosis, intraoperative biopsy culture was reported to be the most accurate test in terms of microbiological diagnosis [18,19,20]. In previous studies, the sensitivity of preoperative synovial fluid cultures and intraoperative biopsy cultures was reported to be 45–75% [21,22] and 65–94% [18,23], respectively. However, a considerably wide range (52–78%) has been reported for the concordance rate between the two tests [12,13,14]. In the present study, the total concordance rate between preoperative and intraoperative cultures was 57% in all three groups (knee, hip and shoulder) of patients with PJI, which was consistent with the literature. The number of studies investigating the said concordance has risen recently due to the polymicrobial infections encountered, contamination issues and other such problems [5,7]. These studies present the overall concordance values rather than classifying patients with PJI according to the joints involved [5,24]. In this study, each group was separately evaluated, and the highest concordance rate was observed in the knee group (60%), followed by the shoulder (56%) and hip (54%) groups. Considering previous studies on concordance, a concordance rate of 78% implies that there is no concordance between preoperative and intraoperative evaluations in one of three to four patients. This may lead to a change in treatment efficacy amongst different patients. Current guidelines recommend using leukocyte count and percentage of polymorphonuclear neutrophils in combination with preoperative synovial fluid aspiration cultures in the diagnosis of PJI. According to the results of this study, although different joints had similar results, concordance between preoperative and intraoperative cultures was observed in nearly one of two patients. Therefore, clinicians should not base postoperative treatment strategies solely on preoperative cultures.

Other studies on this topic lack clear elucidation of essential information such as antibiotic use prior to sampling as well as standardization of synovial fluid and tissue cultures. On the other hand, this study complied with the standard aspiration procedures practiced at our clinic, which made it possible to collect accurate patient data. Current studies show that *Staphylococcus* was the most frequently detected organism according to the microbiological profile of both preoperative aspiration and intraoperative cultures in patients with knee and hip PJI [25,26,27,28,29]. Moreover, *Propionibacterium acnes* was the most commonly reported microorganism in studies investigating PJI of the shoulder [30,31,32]. In this study, *Staphylococcus* was the most commonly detected microorganism in the knee and hip groups, and *Propionibacterium acnes* was the most commonly detected microorganism in the shoulder group, both of which were consistent with the literature.

This study also demonstrated that the rates of high-virulence and difficult-to-treat bacteria were significantly higher in patients with knee and hip PJI compared to the shoulder group. Similarly, the literature also showed that the rate of difficult-to-treat bacteria was lower and low-virulence bacteria were more common in patients with PJI of the shoulder compared to those with hip and knee PJI [32,33].

This study had some limitations. Firstly, despite meticulous methodology, important data could have been missed as a result of the retrospective nature of this study. In addition, although this study included a relatively large patient population, not all relationships of interest could be evaluated as different groups were considered collectively.

## 4. Materials and Methods

### 4.1. Study Design and Patients

This retrospective study used data from a longitudinally maintained institutional database of patients with PJI after obtaining institutional review board approval (2022-300155-WF). This study was performed at a high-volume tertiary referral arthroplasty centre. We retrospectively reviewed patients who underwent septic revision hip, knee and shoulder arthroplasty between January 2016 and December 2019 at our high-volume arthroplasty centre. We included the first 50 consecutive patients that fulfilled the inclusion criteria. We included the preoperative synovial fluid culture and intraoperative tissue culture data for each group (knee, hip and shoulder). Joint aspirations were performed for at least 2 weeks of antibiotic-free follow-up and less than 90 days before surgery; in addition, a minimum of 5 intraoperative tissue samples were taken during revision surgery. Patients with PJI and preoperative dry aspiration or without sufficient culture data were excluded. A total of 150 patients, with 50 patients in each joint group (hip, knee and shoulder PJI), were analysed for this study. PJI was diagnosed according to the Musculoskeletal Infection Society (MSIS) criteria established in 2013 [34] when one major criterion or at least three of the five minor criteria were met. Demographic data, baseline characteristics and body mass index (BMI) were recorded for each patient. The detected microorganisms from both preoperative and intraoperative cultures were classified according to their virulence as high virulence (*Staphylococcus aureus* (*MSSA*) including methicillin-resistant *S. Aureus* (*MRSA*), *Streptococcus species*, *Pseudomonas aeruginosa* and *Enterococci species*) or low virulence (coagulase-negative *Staphylococci* (*CoNS*), *Cutibacterium acnes* and *Escherichia coli*) [33,35]. Furthermore, we classified the microorganisms as difficult to treat (DTT) and not difficult to treat according to the sensitivity to biofilm-active antibiotics [36]. Quinolone-resistant Gram-negative bacteria, rifampicin-resistant *Staphylococcus*, *Enterococcus* and *Candida* were classified as “difficult to treat” (DTT) [37]. Methicillin-resistant *Staphylococci* were abbreviated as “MRS”, and all other organisms (including culture-negative infections and polymicrobial infection without the above-mentioned MRS or DTT) were classified as “easy to treat” (ETT) [36,37,38].

### 4.2. Diagnostic Tests

All preoperative synovial fluid aspirations were performed under strict sterile conditions by a trained physician in a specially designated room. Antibiotics were discontinued two weeks prior to the aspiration. Intraoperative cultures were obtained prior to the administration of perioperative antibiotics. Intraoperative samples were taken during septic knee, hip or shoulder revision surgery. Five to eight tissue samples were collected from multiple surgical sites. All samples were collected in thioglycolate culture bottles and held for culture for 14 days. After this period or at the time of detection of bacterial growth, samples were transferred onto two Columbia blood plates which were incubated at 37 °C under aerobic (5% carbon dioxide) and anaerobic conditions. Speciation was determined by mass spectrometry (matrix-assisted laser desorption ionization and time-of-flight spectroscopy, MALDI-ToF, Bruker, Bremen, Germany). Antimicrobial susceptibility testing was performed using the Vitek-2 system (BioMerieux, Nürtingen, Germany) and gradient agar diffusion Epsilometer tests (Liofilchem, Roseto degli Abruzzi, Italy) in select cases as appropriate. The microbiological diagnostic procedures were carried out in an accredited laboratory (DIN EN ISO 15189). Intraoperative polymicrobial cultures were defined by the presence of bacteria in at least two samples; otherwise, the microorganism found in only one culture was considered contamination. Some authors also used a threshold of two specimens yielding indistinguishable microorganisms in order to increase the sensitivity [39,40], and this is included in the major criteria of PJI consensus documents [34].

### 4.3. Outcome Measures

First, the study cohort was divided into two groups according to the concordance between preoperative cultures and intraoperative sample cultures. Intraoperative cultures were considered definitive. The concordant group included patients who had the same bacteria in both groups. For polymicrobial samples, the same bacteria needed to be present in both the preoperative and intraoperative cultures to be included in the concordant group. The discordant group had at least one different pathogen. Second, we analysed the three groups of 50 patients according to the involved joint, comparing them in terms of demographic data and concordance rate in the different joints. We then analysed and compared the three different cohorts (hip, knee and shoulder) in order to evaluate if, in different joints, there were differences in microorganism patterns in preoperative and intraoperative cultures, according to the type of bacteria (Gram−, Gram+ and polymicrobial), high/low virulence and difficulty to treat. The aim of analyzing bacterial patterns was to assess if there is a need for different empirical antibiotic or therapeutic strategies for different joints.

### 4.4. Statistical Analysis

Statistical analysis was performed using SPSS software (Version 23.0, SPSS Inc., Chicago, IL, USA). Normally distributed continuous variables were expressed with mean ± standard deviation (*p* > 0.05) in Kolmogorov–Smirnov test or Shapiro–Wilk test (*n* < 30) values, and continuous variables without a normal distribution were expressed with median values. The continuous variables were compared with the use of one-way ANOVA or the Kruskal–Wallis test depending on parametric or nonparametric values, respectively. The categorical variables were compared between the groups using the chi-square test or Fisher’s exact test. Pre–post measures data were analysed with McNemar’s test. *p* < 0.05 was considered statistically significant.

## 5. Conclusions

We demonstrated a concordance rate of 56.7% between preoperative synovial fluid and intraoperative tissue cultures in patients with PJI of the knee, hip and shoulder with no significant difference between the three groups. *Staphylococci* and *CoNS* were the most common organisms and demonstrated a high rate of concordance, while polymicrobial infections were highly discordant. Hip and knee PJIs demonstrated a higher rate of high-virulence and difficult-to-treat organisms compared to shoulder PJIs, and this should be considered when formulating an empirical antibiotic and treatment plan. Overall, a modest concordance rate of 56.7% should alert the clinician to not solely rely on preoperative culture results and to wait for definitive intraoperative culture results before instituting a final antibiotic and management plan to maximise treatment success.

## Figures and Tables

**Table 1:** Intraoperative culture (considered the gold standard) comparison between concordant and discordant groups.

Demographic Information and Organism Profile	Total	Concordant _1_	Discordant _2_	*p*_1&2_ Value
Patients (*n*)	150	85 (56.7%)	65 (43.3%)	
Age (years)	69.21 ± 11.71	67.31 ± 11.65	71.69 ± 11.40	0.011 *
BMI	30.6 ± 12.98	31.78 ± 16.63	29.08 ± 5.04	0.317
Sex				
Male	80 (53.3%)	43 (50.6%)	37 (56.9%)	
Female	70 (46.7%)	42 (49.4%)	28 (43.1%)	0.441
Side				
Right	80 (53.3%)	46	34	
Left	70 (46.7%)	39	31	0.826
Organism				
*Staphylococci*	52	42	10	0.0001 *
*MRSA*	0	0	0	1
*MSSA*	13	9	4	0.394
*CoNS*	39	33	6	0.0004 *
*Enterococcus*	2	2	1	1
*Streptococcus*	4	4	0	0.133
*Propionibacterium acnes*	31	21	10	0.162
Other Gram positive	3	0	3	0.079
Gram negative	7	7	0	0.019 *
Polymicrobial	36	2	34	<0.0001 *
Culture negative	14	7	7	0.597

_1_ Concordant group, _2_ Discordant group, * Bold indicates statistical significance.

**Table 2:** A general comparison of the three groups.

	Hip ^1^		Knee ^2^		Shoulder ^3^				
	Mean ± SD	Median (Min.–Max.)	Mean ± SD	Median (Min.–Max.)	Mean ± SD	Median (Min.–Max.)	*p*_1&2_	*p*_1&3_	*p*_2&3_
Age at time of operation (years)	70.5 ± 11.9	72.6 (40–92.7)	69.7 ± 11.6	69.9 (43–88.4)	67.5 ± 11.7	69.2 (44.3–89.3)	0.909	0.391	0.611
Height (cm)	169.3 ± 14.4	170 (100–196)	172.7 ± 9.1	171 (151–190)	171.4 ± 9.1	170 (151–188)	0.290	0.641	0.838
Weight (kg)	90.7 ± 23.4	85 (48–173)	87.3 ± 19.3	83 (54–139)	87.0 ± 19.9	82 (55–139)	0.700	0.659	0.998
BMI	30.1 ± 5.9	30.3 (17.3–44.9)	29.2 ± 5.6	29.0 (19.0–41.5)	29.4 ± 5.5	29.9 (19.0–41.5)	0.669	0.809	0.970
Sex	*n*	%	*n*	%	*n*	%			
Male	27	54.0	27	54.0	26	52.0			
Female	23	46.0	23	56.0	24	48.0	1.00	1.00	1.00
Side									
Right	32	64.0	25	50.0	24	48.0			
Left	18	36.0	25	50.0	26	52.0	0.225	0.158	1.00
Correct identification of bacteria with aspiration									
No	23	46.0	20	40.0	22	44.0			
Yes	27	54.0	30	60.0	28	56.0	0.544	0.841	0.685

Post hoc analysis was not performed because there was no statistically significant difference between the groups. ^1^ Hip Group; ^2^ Knee Group; ^3^ Shoulder Group, BMI: Body mass index, SD: Standard deviation, *n*: number of patient, *p*: *p* value.

**Table 3:** Intraoperative and preoperative comparison of the three groups.

	Hip ^1^		Knee ^2^		Shoulder ^3^					
	*n*	%	*n*	%	*n*	%	*p*	*p*_1&2_	*p*_1&3_	*p*_2&3_
Intraoperative										
Virulence										
Low virulence	28	56.0	30	60.0	44	88.0	**0.0004**	0.074	**0.0011**	**0.056**
High virulence	19	38.0	11	22.0	4	8.0				
Culture negative	3	6.0	9	18.0	2	4.0				
Gram										
Gram−	2	4.0	1	2.0	4	8.0	0.088	0.104	0.662	0.070
Gram+	29	58.0	32	64.0	32	64.0				
Polymicrobial	16	32.0	8	16.0	12	24.0				
Culture negative	3	6.0	9	18.0	2	4.0				
Difficult to treat										
No	39	78.0	36	72.0	47	94.0	**0.030**	0.148	**0.040**	**0.013**
Yes	8	16.0	5	10.0	1	2.0				
Culture negative	3	6.0	9	18.0	2	4.0				
Preoperative										
Virulence										
Low virulence	24	48.0	30	60.0	38	76.0	**0.002**	**0.029**	**0.003**	0.225
High virulence	17	34.0	6	12.0	4	8.0				
Culture negative	9	18.0	14	28.0	8	16.0				
Gram										
Gram−	3	6.0	1	2.0	3	6.0	0.214	0.246	0.246	0.364
Gram+	32	64.0	34	68.0	38	76.0				
Polymicrobial	6	12.0	2	2.0	1	2.0				
Culture negative	9	18.0	14	28.0	8	16.0				
Difficult to treat										
No	36	72.0	33	66.0	41	82.0	0.234	0.423	0.217	0.173
Yes	5	10.0	3	6.0	1	2.0				
Culture negative	9	18.0	14	28.0	8	16.0				

*p*: Chi-square test, *n*: Number of patient, bold indicates statistical significance, ^1^ Hip Group; ^2^ Knee Group; ^3^ Shoulder.

**Table 4:** Intraoperative and preoperative results from the three groups.

	Intraoperative		Preoperative		*p*
	*n*	%	*n*	%	
Hip					
Virulence					
Low virulence	28	56.0	24	48.0	0.181
High virulence	19	38.0	17	34.0	
Culture negative	3	6.0	9	18.0	
Gram					
Gram−	2	4.0	3	6.0	**0.048**
Gram+	29	58.0	32	64.0	
Polymicrobial	16	32.0	6	12.0	
Culture negative	3	6.0	9	18.0	
Difficult to treat					
No	39	78.0	36	72.0	0.148
Yes	8	16.0	5	10.0	
Culture negative	3	6.0	9	18.0	
Knee					
Virulence					
Low virulence	30	60.0	30	60.0	0.278
High virulence	11	22.0	6	12.0	
Culture negative	9	18.0	14	28.0	
Gram					
Gram−	1	2.0	1	2.0	0.086
Gram+	32	64.0	34	68.0	
Polymicrobial	8	16.0	1	2.0	
Culture negative	9	18.0	14	28.0	
Difficult to treat					
No	36	72.0	33	66.0	0.423
Yes	5	10.0	3	6.0	
Culture negative	9	18.0	14	28.0	
Shoulder					
Virulence					
Low virulence	44	88.0	38	76.0	0.132
High virulence	4	8.0	4	8.0	
Culture negative	2	4.0	8	16.0	
Gram					
Gram−	4	8.0	3	6.0	**0.003**
Gram+	32	64.0	38	76.0	
Polymicrobial	12	24.0	1	2.0	
Culture negative	2	4.0	8	16.0	
Difficult to treat					
No	47	94.0	41	82.0	0.134
Yes	1	2.0	1	2.0	
Culture negative	2	4.0	8	16.0	

*n*: number of patient, *p*: Chi-square test, bold indicates statistical significance.

## Data Availability

Data and materials are available, consent to participate was given, consent to publish was given.
